# Are Neutrophil-Lymphocyte, Platelet-Lymphocyte, and Monocyte-Lymphocyte Ratios Prognostic Indicators in Patients With Chronic Obstructive Pulmonary Disease in Intensive Care Units?

**DOI:** 10.7759/cureus.23499

**Published:** 2022-03-26

**Authors:** Guler Eraslan Doganay, Mustafa Ozgur Cirik

**Affiliations:** 1 Anesthesiology and Reanimation, Ataturk Chest Diseases and Thoracic Surgery Training and Research Hospital, Ankara, TUR

**Keywords:** intensive respiratory care, copd: chronic obstructive pulmonary disease, monosit to lymphocyte ratio(mlr), platelet to lymphocyte ratio (plr), neutrophil to lymphocyte ratio (nlr)

## Abstract

Background and objective

Chronic obstructive pulmonary disease (COPD) is a condition in which the expiratory airflow is restricted and is characterized by inflammation. Recently, inflammation-related biomarkers such as neutrophil-lymphocyte ratio (NLR), platelet-lymphocyte ratio (PLR), and monocyte-lymphocyte ratio (MLR) have been used to predict the prognosis in COPD. The aim of this study was to evaluate the role of biomarkers such as NLR, PLR, and MLR in COPD patients in intensive care and to examine the ability of these markers to predict the prognosis [length of stay in hospital (LOSH), duration of mechanical ventilation (MV), length of stay in ICU (LOS ICU), and mortality].

Methods

A total of 562 patients who were treated in the ICU between 2018 and 2019 were retrospectively reviewed. Among them, 369 were patients with COPD. We evaluated clinical data including patient demographics, Charlson Comorbidity Index (CCI), Acute Physiology and Chronic Health Evaluation II (APACHE II) score, Sequential Organ Failure Assessment (SOFA) score, LOS ICU, LOSH, duration of MV, as well as NLR, PLR, and MLR values. Data on patient deaths (30-day mortality) was obtained from the Death Notification System.

Results

Age, LOSH, CCI, and SOFA were found to predict mortality in COPD patients. In cases with mortality, age, inotropic use, MV duration, LOS ICU, APACHE II, CCI, SOFA, lymphocyte count, neutrophil count, platelet count, monocyte count, NLR, PLR, and MLR levels were statistically significantly higher than those in cases without mortality. There was a positive and low statistically significant relationship of NLR, PLR, and MLR with prognostic factors like MV duration, APACHE II scores, and SOFA scores.

Conclusion

The NLR, PLR, and MLR values may be used as prognostic indicators in COPD patients in intensive care. Although there are many studies endorsing the use of biomarkers such as NLR, PLR, and MLR as prognostic indicators, further comparative studies on this subject are still required to gain deeper insights into the topic.

## Introduction

Chronic obstructive pulmonary disease (COPD) is a condition where the expiratory airflow is restricted and is characterized by inflammation. Although many systemic biomarkers are used in COPD, recently, neutrophil-lymphocyte ratio (NLR), monocyte-lymphocyte ratio (MLR), and platelet-lymphocyte ratio (PLR), which can be calculated from the routine complete blood count, have come to be widely used to predict the prognosis due to their low cost and easy availability [1,2]. The degree of pulmonary inflammation in COPD is strongly related to smoking [3]. In various studies, NLR was reported to be high in patients with COPD exacerbation. Comorbidities and extrapulmonary symptoms also affect the survival and severity of disease in COPD patients. Many comorbidities are also associated with inflammation [4,5]. Acute exacerbation of COPD (AECOPD) is characterized by more severe inflammation compared to stable patients and may increase the length of stay in hospital (LOSH) and length of stay in ICU (LS ICU), and may also require more invasive treatments such as mechanical ventilation (MV) [6,7].

Previous studies on NLR and COPD have shown that NLR affects airflow limitation, the severity of the disease, exacerbation, LOSH, and mortality [8-11]. The aim of this study was to evaluate the role of biomarkers such as NLR, PLR, and MLR in COPD patients in intensive care and to investigate the ability of these markers to predict the prognosis (LOSH, duration of MV, LS ICU, and mortality).

## Materials and methods

After obtaining ethical approval from the Medical Specialization Training Board of Ataturk Chest Diseases and Thoracic Surgery Training and Research Hospital (15/10/2020-697), 562 patients treated in the pulmonary ICU during the two-year period between 2018 and 2019 were retrospectively reviewed. Of them, 369 had a history of COPD. We evaluated clinical data including patient demographics, Charlson Comorbidity Index (CCI), Acute Physiology and Chronic Health Evaluation II (APACHE II) scores, Sequential Organ Failure Assessment (SOFA) scores, LOS ICU, LOSH, duration of MV, as well as NLR, PLR, and MLR values. Data on patient deaths (30-day mortality) was obtained from the Death Notification System.

The inclusion criteria were as follows: all COPD patients who needed non-invasive or invasive MV in the ICU. Patients with severe lung diseases such as asthma, bronchiectasis, tuberculosis, and malignancy were excluded from the study.

Statistical methods

The results were compared using SPSS Statistics version 22.0 (IBM, Armonk, NY). Whether the distribution of continuous variables was normal or not was determined by the Kolmogorov-Smirnov test. Continuous data were described as mean ± SD and median (interquartile range) for skewed distributions. Categorical data were presented as numbers and percentages. Categorical variables were compared using Pearson’s chi-squared test or Fisher’s exact test.

Firstly, possible risk factors that were thought to be related to mortality were analyzed via one variable multinomial logistic regression analysis. Variables with p<0.25 in the univariate logistic regression analysis were included in multivariate logistic regression analysis. The Backward Wald method was used for multivariate logistic regression analysis. The receiver operating characteristic (ROC) curve analysis was used to determine the cut-off points. A p-value <0.05 was considered statistically significant and a p-value between 0.05 and 0.10 was deemed to be borderline significant level on all statistical analyses.

## Results

In order to determine the factors affecting mortality in COPD patients, logistic regression analysis of single variables was performed first (univariate analysis). Variables with p<0.25 in the univariate logistic regression analysis were included in multivariate logistic regression analysis. The Backward Wald method was used for multivariate logistic regression analysis. The results of step 5, which is the last step of the analysis, are given in Table 1. According to the results, it was determined that age, LOSH, CCI, and SOFA affected mortality in COPD patients.

**Table 1:** The factors affecting mortality in COPD patients Multinomial logistic regression nagelkerke R^2^=0.512 (Hosmer-Lemeshow p>0.05) COPD: chronic obstructive pulmonary disease; OR: odds ratio; LOSH: length of stay in hospital; LOS ICU: length of stay in intensive care unit; MV: mechanical ventilation; APACHE II: Acute Physiology and Chronic Health Evaluation II; CCI: Charlson Comorbidity Index; SOFA: Sequential Organ Failure Assessment

Variables (n=369)	Univariate analysis					Multivariate analysis (Backward Wald 5th step)				
	Wald	P-value	OR	95% CI for EXP(B)		Wald	P-value	OR	95% CI for EXP(B)	
				Lower	Upper				Lower	Upper
Age	7.332	0.007	1.027	1.007	1.047	8.232	0.004	1.065	0.942	0.989
Gender	0.306	0.580	1.132	0.729	1.758					
Malignancy	1.296	0.255	0.607	0.257	1.434					
LOSH	2.042	0.153	0.989	0.975	1.004	4.427	0.035	1.015	1.001	1.030
LOS ICU	9.141	0.002	1.056	1.019	1.095					
MV duration	20.847	<0.001	1.111	1.062	1.163					
APACHE II score	45.444	<0.001	1.130	1.090	1.170					
CCI	19.046	<0.001	1.295	1.153	1.455	6.230	0.013	1.207	1.041	1.400
SOFA score	67.431	<0.001	1.751	1.532	2.001	4.571	0.033	1.114	1.009	1.230
Neutrophil-lymphocyte ratio	0.436	0.509	0.998	0.990	1.005					
Platelet-lymphocyte ratio	0.484	0.487	1.000	1.000	1.001					
Monocyte-lymphocyte ratio	0.352	0.553	0.972	0.883	1.069					

The ROC curve analysis was applied to determine the success of NLR, PLR, and MLR in predicting mortality and to give the cut-off value for mortality in COPD patients. It showed that it could distinguish between NLR cases in determining the risk of mortality; that is, it can correctly classify the patients at a rate of 63%, 61.4%, and 63.4% (intermediate level), respectively. In order to answer the question as to which value should be taken as the cut-off value for this test, the sensitivity and specificity values ​​obtained as a result of the analysis were examined and the optimum point was selected.

**Figure 1:**
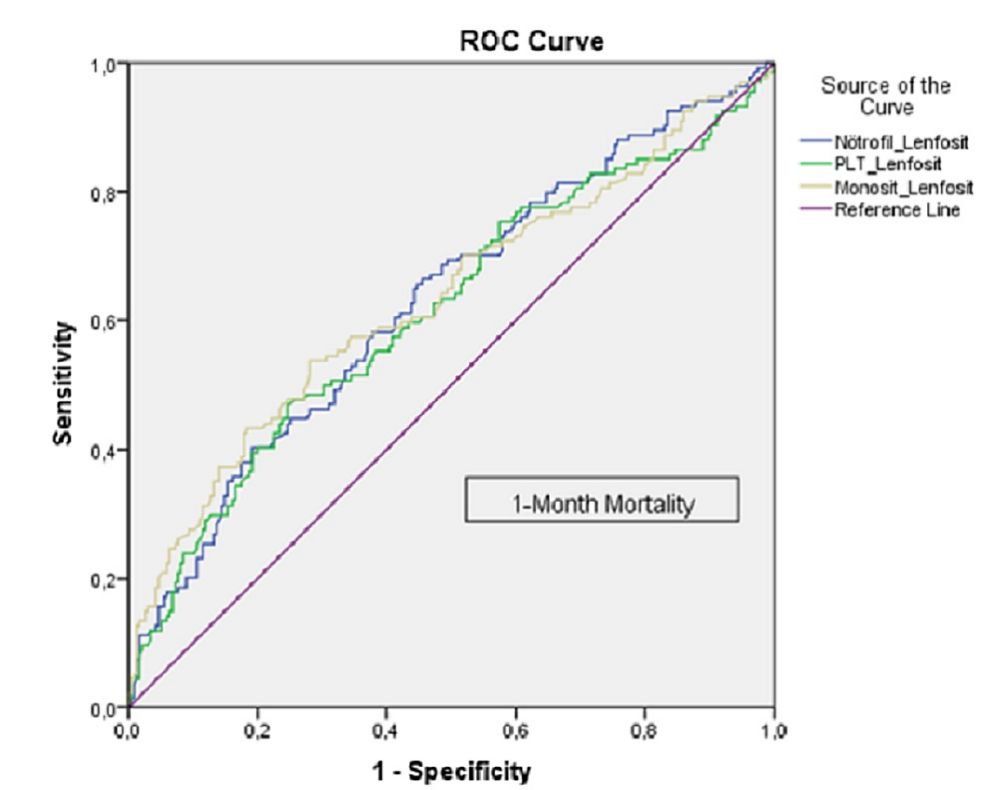
ROC curve analysis ROC: receiver operating characteristic

The sensitivity value for NLR was 40.3%, the specificity value was 80.9%, and the cut-off value was 21.71. As a result, the risk of mortality was higher in cases with NLR 21.71 and above. The sensitivity value for PLR was 47%, the specificity value was 75.3%, and the cut-off value was 423.54. As a result, the risk of mortality was higher in cases with PLR 423.54 and above. The sensitivity value was 53.7% for MLR, the specificity value was 71.9%, and the cut-off value was 0.873. As a result, the risk of mortality was higher in cases with MLR 0.873 and above (Table 2). Figure 1 also presents the ROC curve analysis.

**Table 2:** ROC curve analysis for predicting mortality ROC: receiver operating characteristic; AUC: area under the curve

Test variables	AUC	P-value	95% CI		Cut-off	Sensitivity	Specificity
			Lower	Upper			
Neutrophil-lymphocyte ratio	0.630	<0.001	0.570	0.689	21.71	40.3%	80.9%
Platelet-lymphocyte ratio	0.614	<0.001	0.552	0.676	423.54	47%	75.3%
Monocyte-lymphocyte ratio	0.634	<0.001	0.573	0.695	0.873	53.7%	71.9%

As per the comparison between the cases with mortality and those without mortality, age, inotropic use, MV duration, LOS ICU, APACHE II, CCI, SOFA, lymphocyte count, neutrophil count, platelet count, monocyte count, NLR, PLR, and MLR levels were found to be statistically significantly higher in the mortality group (Table 3).

**Table 3:** The comparison between cases with mortality and those without mortality Continuous variables are expressed as mean ± standard deviation (SD) and median (IQR); categorical variables are expressed as frequency and percentage. Continuous variables were compared using the Mann-Whitney U test and categorical variables were compared using Pearson’s chi-square test or Fisher exact test LOSH: length of stay in hospital; LOS ICU: length of stay in intensive care unit; MV: mechanical ventilation; APACHE II: Acute Physiology and Chronic Health Evaluation II; CCI: Charlson Comorbidity Index; SOFA: Sequential Organ Failure Assessment

Variables (n=369)			Mortality (n=126)			No mortality (n=243)			P-value
Gender, n (%)	Male		86 (64.2%)			144 (61.3%)			0.580
	Female		48 (35.8%)			91 (38.7%)			
Inotropic use, n (%)			70 (52.2%)			18 (7.7%)			<0.001
Malignancy, n (%)			12 (9%)			14 (6%)			0.279
Age		74.22		±11.23	75 (17)	70.87	±11.26	71 (17)	0.003
MV duration			5.25	±7.24	3 (2)	1.68	±5.19	0 (4)	<0.001
LOS ICU			7.03	±6.89	4.5 (4)	4.90	±5.67	3 (7)	0.003
LOSH			18.84	±13.26	17 (15)	21.23	±16.40	16 (16)	0.235
APACHE II score			25.55	±8.00	24 (7)	19.59	±6.05	19 (11)	<0.001
CCI			6.67	±2.09	7 (3)	5.72	±1.79	6 (3)	<0.001
SOFA score			7.34	±2.68	7 (2)	4.88	±1.56	4 (4)	<0.001
Lymphocyte count			0.97	±1.18	0.7 (0.8)	1.09	±0.89	0.9 (0.9)	0.008
Neutrophil count			12.62	±7.69	11 (7.5)	9.76	±6.31	8.4 (6.2)	<0.001
Platelet count			275.40	±138.18	243.5 (148.5)	238.68	±101.71	222 (113)	0.041
Monocyte count			0.93	±0.80	0.78 (0.89)	0.79	±1.20	0.5 (0.58)	<0.001
Neutrophil-lymphocyte ratio			25.94	±30.96	15 (20.32)	16.43	±30.00	9.44 (13.99)	<0.001
Platelet-lymphocyte ratio			581.28	±616.14	387.71 (504.25)	365.60	±361.78	258.33 (268.72)	<0.001
Monocyte-lymphocyte ratio			2.06	±3.10	0.90 (1.69)	0.93	±1.44	0.58 (0.7)	<0.001

Spearman correlation analysis was applied to determine the relationship of NLR, PLR, and MLR with other variables of 369 COPD patients in the ICU, and the results are presented in Table 4. Based on Spearman correlation analysis, there was a positive and low statistically significant correlation between NLR and MV duration as well as APACHE II and SOFA scores. There was also a positive and low statistically significant relationship between PLR and MV duration. We also found a positive and low statistically significant relationship between MLR and MV duration as well as APACHE II and SOFA scores.

**Table 4:** The correlation of NLR, PLR, and MLR with other variables NLR: neutrophil-lymphocyte ratio; PLR: platelet-lymphocyte ratio; MLR: monocyte-lymphocyte ratio; LOSH: length of stay in hospital; LOS ICU: length of stay in intensive care unit; MV: mechanical ventilation; APACHE II: Acute Physiology and Chronic Health Evaluation II; CCI: Charlson Comorbidity Index; SOFA: Sequential Organ Failure Assessment

Variables		NLR	PLR	MLR
Age	r	0.041	-0.011	0.030
	p	0.427	0.837	0.570
	n	369	369	369
LOSH	n	0.032	0.016	0.059
	p	0.544	0.760	0.256
	n	369	369	369
LOS ICU	r	0.078	0.060	0.087
	p	0.137	0.247	0.097
	n	369	369	369
MV duration	r	0.144	0.117	0.104
	p	0.006	0.024	0.046
	n	369	369	369
APACHE II score	r	0.116	0.023	0.108
	p	0.026	0.660	0.039
	n	368	368	368
CCI	r	0.025	-0.002	0.031
	p	0.628	0.964	0.553
	n	369	369	369
SOFA score	r	0.125	0.038	0.108
	p	0.016	0.465	0.037
	n	369	369	369

## Discussion

This study was conducted to evaluate the utility of NLR, PLR, and MLR as prognostic indicators in patients with COPD. Our findings showed that age, LOSH, CCI, and SOFA affected mortality in COPD patients in ICU. Additionally, in cases with mortality, lymphocyte count, neutrophil count, platelet count, monocyte count, NLR, PLR, MLR were statistically significantly higher than in cases without mortality. Our study also revealed that there was a positive correlation of both NLR and MLR with MV duration, APACHE II scores, and SOFA scores. There was also a positive correlation between PLR and MV duration.

The utility of NLR, PLR, and MLR as prognostic markers has been a topic of controversy in various studies. In 2014, Günay et al. [12] suggested that NLR could be accepted as a new inflammatory marker for COPD patients, and many studies have shown that NLR is associated with airflow limitation, exacerbation, hospitalization, and mortality [13,14]. Lee et al. [10] suggested that NLR may potentially serve as a biomarker in COPD exacerbation because NLR was significantly higher in patients with COPD exacerbation who required hospitalization. This is in line with our findings as we found a correlation of NLR with MV duration, APACHE II scores, and SOFA scores. Lou et al. suggested that elevated NLR and PLR predicted an increased risk of 28-day mortality in patients with AECOPD. However, MLR failed to show any prognostic significance [15]. Controversially, Aksel found that both NLR and PLR are not suitable as prognostic markers in terms of poor clinical outcomes and mortality in COPD exacerbation [16]. Sakurai et al. [14] showed the appropriate cut-off value of NLR to be 2.7 to predict COPD severity and future exacerbations. In our study, we found the cut-off value for predicting mortality to be 2.71.

In patients with COPD, inflammation in the lungs may increase the activation of neutrophils [17]. The NLR value is obtained by dividing the absolute neutrophil count by the absolute lymphocyte count and the test is fairly inexpensive and can be used for screening patients [18]. Some studies have reported that a high PLR is associated with poor prognosis in many physiological stress conditions, especially neonatal sepsis and malignancy [19,20]. In our study, we found a positive and low statistically significant relationship between PLR and MV duration.

Additionally, MLR has recently been used as an indicator of inflammation, morbidity, and mortality [21,22]. Consistent with previous studies on diseases in which inflammation plays an important role in the pathophysiology, such as cardiovascular disease and psoriasis, increased MLR was found to be associated with disease severity [23]. In our study, we found a positive and low statistically significant relationship of MLR with MV duration, APACHE II scores, and SOFA scores.

Limitations of our study include its retrospective nature and single-center design. The present results were derived from short-term follow-ups. We recommend further studies focusing on long-term outcomes to better analyze the topic.

## Conclusions

Based on our findings, NLR, PLR, and MLR may be used as prognostic indicators in patients with COPD in intensive care. Although many studies have already endorsed the use of biomarkers such as NLR, PLR, and MLR as prognostic indicators, further comparative studies on this subject are required to arrive at more definitive findings.
